# Bayesian sample size determination for longitudinal intervention studies with linear and log-linear growth

**DOI:** 10.3758/s13428-025-02749-5

**Published:** 2025-07-28

**Authors:** Ulrich Lösener, Mirjam Moerbeek

**Affiliations:** Department of Methodology and Statistics, Utrecht University, Utrecht, Netherlands

**Keywords:** Sample size determination, Multilevel model, Longitudinal data, Bayes factor, Power, Monte Carlo simulation, Log-linear

## Abstract

A priori sample size determination (SSD) is essential for designing cost-efficient trials and in avoiding underpowered studies. In addition, reporting a solid justification for a certain sample size is required by most ethics committees and many funding agencies. Often, SSD is based on null hypothesis significance testing (NHST), an approach that has received severe criticism in the past decades. As an alternative, Bayesian hypothesis evaluation using Bayes factors has been developed. Bayes factors quantify the relative support in the data for a pair of competing hypotheses without suffering from some of the drawbacks of NHST. SSD for Bayesian hypothesis testing relies on simulations and has only been studied recently. Available software for this is limited to simple models such as ANOVA and the *t* test, in which observations are assumed to be independent from each other. However, this assumption is rendered untenable in longitudinal experiments where observations are nested within individuals. In that case, a multilevel model should be used. This paper provides researchers with a valuable tool for performing SSD for multilevel models with longitudinal data in a Bayesian framework, along with the necessary theoretical background and concrete empirical examples. The open-source R function that enables researchers to tailor the simulation to their trial at hand can be found on the GitHub page of the first author.

## Introduction

An integral part of planning an experiment is determining the necessary sample size to achieve a desired level of statistical power. Power is the probability of finding an existing effect, which is equivalent to the probability of *not* committing a type II error. A type II error occurs when falsely concluding that there is no effect present (false negative). The power of a trial depends on the sample size, effect size, as well as the probability of making a type I error (falsely concluding that there is an effect; Cohen, 2013). Researchers strive for high power in order to have high chances of finding an existing effect and often search for the sample size necessary to achieve a desired power level. This procedure is referred to as “sample size determination” (SSD) and it is required by most ethical committees and many funding agencies. The benefit of SSD is twofold: On the one hand, it serves to avoid underpowered studies where the hypothesized effect may exist in the population but the researcher fails to detect it because their sample size is too small (type II error). Underpowered studies remain a major problem in psychological science (Maxwell, 2004; Vadillo, Konstantinidis, & Shanks, 2016). On the other hand, SSD can avoid overpowered studies with unnecessarily large sample sizes, which are inefficient and wasteful. Additionally, they can be problematic because small, practically insignificant differences become statistically significant, potentially leading to flawed and overconfident conclusions (Faber & Fonseca, 2014; Kaplan, Chambers, & Glasgow, 2014). In both cases of inadequate sample size, resources are wasted and unnecessary strain is put on participants (Case & Ambrosius, 2007), resulting in unethical research practice.

Following widespread criticism of the frequentist approach to null hypothesis significance testing (NHST) using *p* values, Bayesian hypothesis evaluation employing the Bayes factor (BF) has been developed as an alternative inferential tool (Jeffreys, 1935; Kass & Raftery, 1995). The use of BFs is rapidly gaining popularity among researchers (Schmalz, Biurrun Manresa, & Zhang, 2023; Van De Schoot, Winter, Ryan, Zondervan-Zwijnenburg, & Depaoli, 2017). However, some researchers are still hesitant to use Bayesian methods of hypothesis testing, most likely due to a lack of exposure in applied psychological fields and its seemingly complex nature – an issue that must be addressed by providing user-friendly software and making Bayesian methods more accessible (Van De Schoot et al., 2017). We aim to achieve this by employing the approximate adjusted fractional Bayes factor (AAFBF), which evaluates a pair of competing hypotheses about maximum likelihood estimates from frequentist models (Hoijtink, Mulder, van Lissa, & Gu, 2019). Also, the calculation of the AAFBF is quite straightforward compared to other BFs, and it is not necessary for the user to specify a prior distribution, as the AAFBF relies on a default prior (Berger & Pericchi, 1996). Finally, the fact that the AAFBF does not require repeated sampling from a posterior distribution makes it computationally inexpensive and thus ideal for large-scale simulation studies.

Despite the growing popularity of BFs, the software available for Bayesian SSD is either limited to fully Bayesian model estimation and hypothesis evaluation (e.g., Vasishth, Yadav, Schad, & Nicenboim, 2023) or to simple models such as ANOVA and t test (Fu, 2022). We believe that the former approach neglects the need for accessibility of Bayesian methods, while the latter neglects the fact that in psychological research, statistical models are often more sophisticated. For example, in the presence of longitudinal data, multilevel regression models are the method of choice (Hedeker & Gibbons, 2006). Using multilevel models, we can evaluate the effectiveness of a treatment intervention over time by comparing the mean growth trajectories of an experimental and control condition. The power of such experiments also depends on the number of measurements and their location in time, which is why we cannot simply use the SSD results from a t test or an ANOVA.

In this paper, we address the scarcity of accessible software for Bayesian SSD for trials with longitudinal data with linear or log-linear growth by introducing an open-access R function available on GitHub. This function performs Bayesian SSD for multilevel models via the AAFBF using a Monte Carlo simulation. For now, we focus on the case where two competing hypotheses are formulated about linear or log-linear growth. This aims at extending the work by Fu, Hoijtink, and Moerbeek (2021) to the more complex case of multilevel models with linear as well as log-linear growth. In doing so, we hope to lower the threshold of employing Bayesian hypothesis evaluation in longitudinal experiments. The subsequent sections of this paper are organized as follows. First, we describe the multilevel model used in this paper. Next, we elaborate on Bayesian hypothesis evaluation generally and the AAFBF specifically, followed by a section on our Bayesian SSD algorithm. Subsequently, we illustrate our method using two empirical examples. Finally, we provide a summary of our study results along with directions for future research.

## Multilevel models

Suppose we want to assess the effectiveness of a new psychological treatment intervention on quality of life in an experimental setting with randomized allocation to either the treatment or the control condition. To this end, each individual’s quality of life scores are measured repeatedly over time, meaning that we are dealing with longitudinal data. This is a typical situation in psychological intervention research, for which multilevel models have been implemented for a long time (Bryk & Raudenbush, 1987). The reason why many traditional methods, such as ANOVAs and single-level regression, are not suitable for this type of data is that the assumption of independence of observations is not tenable (Walsh, 1947; Raudenbush & Bryk, 2002). This is because observations belonging to the same individual tend to be more similar than observations across individuals. Employing models that ignore the nesting of observations within individuals can lead to type I or type II error inflation (Moerbeek, van Breukelen, & Berger, 2003). Alternatively, repeated measures ANOVA can be used, but this model relies on strict assumptions such as compound symmetry (i.e., constant covariances over timepoints) and equal spacing of time points (Hox, Moerbeek, & Van de Schoot, 2018). Furthermore, with repeated measures ANOVA, all measurements of an individual need to be excluded from the analysis if there is a missing value on just one occasion. This results in a substantial loss of power when attrition is present. However, these drawbacks do not apply to multilevel models in which the nested structure of the data is explicitly modeled using a hierarchical set of regression equations (Raudenbush & Bryk, 2002; Goldstein & Browne, 2003; Bosker & Snijders, 2011). Additionally, multilevel models allow for the measurements to be irregularly spaced in time. Note, however, that we do assume that measurement occasions do not differ per individual, i.e., that all participants are measured on the same set of points in time. This is because in trials where measurement occasions are not fixed before data collection and vary per subject, the researcher typically does not know when each individual will be measured, making it very difficult to enter this information beforehand. For a comprehensive overview of multilevel models, see the book by Hox et al. (2018). In this paper, we will limit our scope to modeling linear and log-linear growth as they represent popular choices in psychological intervention research (e.g., Althammer, Reis, Van der Beek, Beck, & Michel, 2021). In linear growth models, it is assumed that changes occur at the same rate over time while log-linear models assume that the rate of change becomes smaller over time. Also, we consider the case where measurement occasions are not equally spaced, meaning that the frequency of observation varies across the timeline – a common scenario in longitudinal clinical trials (Gibbons, Hedeker, & DuToit, 2010).

### The two-level model for longitudinal trials

Consider a longitudinal data set consisting of *N* individuals with *n* measurements each, assigned to either a control or an experimental treatment condition. Group membership is indicated by the binary variable $C_i$, where $C_i=0$ for the control group and $C_i=1$ for the experimental group. The points in time of the measurement occasions are represented by the vector $T_j$, which is of length *n*. At the first (within-person) level, the model is defined by the regression equation

1 $$\begin{aligned} Y_{ij} = \pi _{0i} + \pi _{1i}T_j + e_{ij} \qquad \text {with}\ e_{ij} \sim N(0, \sigma ^2), \end{aligned}$$

where $Y_{ij}$ is the outcome score (e.g., quality of life) for individual $i=1,...,N$ at measurement occasion $j=1,...,n$. The person-specific intercept is denoted by $\pi _{0i}$, and $\pi _{1i}$ is the person-specific slope of the time variable $T_j$. The term $e_{ij}$ represents the residual error. The log-linear growth model can be obtained by simply taking the natural logarithm of the time variable ($log(T_j)$). This transformation implies that the growth curve is relatively large at the beginning of the study and levels off over time – a common pattern in psychological intervention studies. At the second (between-person) level, the intercept and slope are modeled as

2 $$\begin{aligned} \pi _{0i}= &  \beta _0 + u_{0i}\end{aligned}$$

3 $$\begin{aligned} \pi _{1i}= &  \beta _1 + \beta _2 C_i + u_{1i}, \end{aligned}$$

where $\beta _0$ is the grand mean intercept and $u_{0i}$ is the individual deviation from that mean. The average slope for the control group is $\beta _1$ while $\beta _2$ captures the difference in slope between the two treatment conditions. The random slope deviation for individual *i* is given by $u_{1i}$. Thus, we expect that some of the variance of the slope $\pi _{1i}$ can be explained by the binary predictor “treatment condition” ($C_i$). The overall intercept $\pi _{0i}$ does not depend on the condition $C_i$ because of the random allocation of subjects to treatment conditions. This means that initial group differences can be attributed to chance and should be minimal, while individual differences may still exist. The population distribution of the random effects $(u_{0i}, u_{1i})^\intercal$ is assumed to be bivariate normal with means of zero $N((0,0)^\intercal , \Sigma _u)$ with the variance-covariance matrix

$$\begin{aligned} \Sigma _u = \begin{pmatrix} \sigma ^2_{u0} & \sigma _{u0u1}\\ \sigma _{u0u1} & \sigma ^2_{u1} \end{pmatrix}. \end{aligned}$$

By substituting (2) and (3) into (1), we obtain the combined regression equation containing both levels:

4 $$\begin{aligned} Y_{ij} = \beta _0 + u_{0i} + (\beta _1 + \beta _2 C_i + u_{1i})T_j + e_{ij} \end{aligned}$$

or, when rearranging the terms:

5 $$\begin{aligned} Y_{ij} = \beta _0 + \beta _1 T_j + \beta _2 C_i T_j + u_{0i} + u_{1i} T_j + e_{ij} \end{aligned}$$

The parameter of interest in this model, when examining a potential treatment effect, is $\beta _2$, indicating the magnitude of interaction between time ($T_j$) and treatment condition ($C_i$). Thus, $\beta _2$ represents the differential growth rate of quality of life across time in the two experimental conditions. If $\beta _2=0$, then individuals in the treatment and control conditions exhibit the same growth/decline in terms of quality of life, implying that no treatment effect is present. If $\beta _2>0$, then individuals in the treatment condition exhibit a larger increase in quality of life, implying that there is a positive treatment effect. Two typical hypotheses on a potential intervention effect can be formulated as follows.

6 $$\begin{aligned} \mathcal {H}_0: \beta _2 = 0\end{aligned}$$

7 $$\begin{aligned} \mathcal {H}_1: \beta _2 > 0 \end{aligned}$$

As shown by Moerbeek and Teerenstra (2015), the standard error of the estimated $\beta _2$ is defined as

8 $$\begin{aligned} \sigma _{\hat{\beta }_2} = \sqrt{\frac{4(\sigma _e^2 + \sigma _{u1}^2 \sum _{j=0}^{n-1}T_j^2)}{N \sum _{j=0}^{n-1}T_j^2}}, \end{aligned}$$

where $T_j$ is the point in time of measurement *j* and *n* is the number of measurement occasions. The fact that the level 2 sample size *N* is in the denominator of Eq. 8 indicates that $\sigma _{\hat{\beta }_2}$ decreases with larger samples. Therefore, our estimation of $\beta _2$ becomes increasingly precise as more individuals are taken into account. This is rather intuitive yet crucial for the logic of SSD: We need to increase *N* until our estimation of $\beta _2$ is sufficiently precise to make a sound inferential decision with respect to our hypotheses. Furthermore, the number of measurements per individual, *n*, is present in both the numerator and the denominator through the summation of squared timepoints. This means that increasing *N* has a greater effect on power compared to increasing *n*. This analytical implication is also reflected in the simulation results presented later in this paper (Table 2). Within the frequentist framework, SSD can be done analytically via closed-form equations relating $\sigma _{\hat{\beta }_2}$ to statistical power (Moerbeek & Teerenstra, 2015). In this paper, however, we employ the BF for which there are no closed-form solutions readily available for SSD, and we therefore rely on simulation (see Fu, 2022).

To visualize the regression slopes for each treatment condition for various combinations of (co-)variance components, we created a Shiny app, which can be found at https://losener.shinyapps.io/MLM_input. This may provide users with some guidance as to the consequences of choosing certain combinations of (co-)variance components and regression parameters on the resulting slopes and uncertainties for linear as well as log-linear growth.

### Three-level models

Sometimes, it is sensible to add a third level of nesting to the model. Suppose that repeated measures are taken within patients that are, in turn, nested within therapists. It is plausible that different therapists have different systematic influences on treatment outcomes. In this case, randomization can occur at either the therapist level or the patient level. When randomizing at the patient level, each patient is randomly allocated to either the control or the experimental condition and is treated by therapists who may also treat patients from the other treatment group. When randomizing at the therapist level, each therapist is randomly allocated to either condition and does not switch conditions during the study. It is often recommended to randomize at the lowest possible level (Moerbeek, 2005). However, when contamination is present, randomization at the therapist level is preferred. Contamination or carry-over effects occur when therapists consciously or unconsciously use techniques from one treatment on a patient who belongs to another treatment group. We created two functions to carry out Bayesian SSD for three-level models with randomization on each level, which can be accessed via the GitHub of the first author (https://github.com/ulrichlosener/BayesianSSD). For an overview of the regression equations for three-level models, see De Jong, Moerbeek, and Van der Leeden (2010)

### Effective sample size in multilevel models

An important issue in Bayesian hypothesis evaluation for multilevel models is the quantification of the effective sample size $N_{eff}$ which quantifies the total amount of information in the data (Berger & Pericchi, 1996). The assumption that $N_{eff}=N_{total}$ (where $N_{total}=N*n$) is invalid, as measurements within individuals are correlated with each other and therefore cannot be counted as independent observations. Thus, $N_{eff}$ should be somewhere between $N_{total}$ and *N*, depending on the degree of correlation of observations within individuals.^1^ The magnitude of this correlation can be expressed in the *intraclass correlation coefficient* (ICC; Killip, Mahfoud, & Pearce, 2004), which is obtained from the intercept-only model. However, because we are using a multilevel model with random slopes, we cannot calculate $N_{eff}$ via the ICC (Sekulovski & Hoijtink, 2023). Instead, we opt for the approach by Faes, Molenberghs, Aerts, Verbeke, and Kenward (2009), where $N_{eff}$ is defined as the sample size needed in an independent sample to achieve the same amount of information as in the actual correlated sample. In this approach, a weight is derived as the ratio of the variance of the estimate under independence of observations and the actual variance of the estimate. Then, $N_{eff}$ is obtained by multiplying the total sample size by this weight.

### Study duration and frequency of observation

In our model, the measurement occasions $j=1,...,n$ are assumed to be the same for each individual (as indicated by the absence of the subscript *i* in $T_j$). However, measurement occasions do not need to be equally spaced in time. This means that the model allows, for example, for more frequent measurements at the beginning and end of an experiment versus halfway through a study. The study duration is denoted by *D*. In the case of equally spaced measurements, the frequency of observation *f* represents the number of measurements taken in each unit time. Assuming that the first measurement is taken at baseline (i.e., time point zero), one can derive the time vector $T_j$ using *D* and *f* as follows: $T_j=((j-1)/f)_{j=1}^D$. Hence, a study with duration *D* and frequency of observation *f* has a total of $n=fD+1$ measurement occasions and the study terminates at time $D=(n-1)/f$ (Raudenbush & Liu, 2001). In case measurements are spaced irregularly, *f* is the average number of measurements per unit time. One of our interests lies in investigating the effect of *f* and *D* on the power of a longitudinal intervention study.

The tool we employ to evaluate $\mathcal {H}_0$ and $\mathcal {H}_1$ is the BF, more specifically, the AAFBF (Gu, Mulder, & Hoijtink,^2018^). The following section offers an introduction to Bayes factors in general and the AAFBF in particular, along with a brief subsection on different types of hypotheses.

## Bayesian hypothesis evaluation via the Bayes factor

In this section, we provide a general overview of Bayesian hypothesis evaluation using the BF in general. Next, we discuss some of its advantages over traditional null hypothesis significance testing (NHST) methods. Finally, we briefly describe the hypotheses used in this paper before elaborating on the specific BF employed in this paper - the AAFBF.

The BF (Kass & Raftery, 1995) quantifies the relative support in the data for a pair of competing hypotheses. It is defined as the ratio of the marginal likelihoods under the two hypotheses. Therefore,

9 $$\begin{aligned} BF_{01}=\frac{m(X \mid \mathcal {H}_0)}{m(X \mid \mathcal {H}_1)} , \end{aligned}$$

where *X* is the data at hand and $m(X \mid \mathcal {H})$ denotes the marginal likelihood under hypothesis $\mathcal {H}$. The prior odds reflect the probability assigned to a hypothesis relative to the probability of the competing hypothesis *before* considering any data. If $\mathcal {H}_0$ is a priori considered to be twice as likely than $\mathcal {H}_1$, then the prior odds would be $\frac{P(\mathcal {H}_0)}{P(\mathcal {H}_1)} = 2$. The posterior odds $\frac{P(\mathcal {H}_0 \mid X)}{P(\mathcal {H}_1 \mid X)}$ denote the updated belief about the relative probability of a hypothesis being true *after* considering the data. The posterior odds are calculated by multiplying the prior odds with the BF.

10 $$\begin{aligned} \frac{P(\mathcal {H}_0 \mid X)}{P(\mathcal {H}_1 \mid X)} = BF_{01} * \frac{P(\mathcal {H}_0)}{P(\mathcal {H}_1)} \end{aligned}$$

Hence, the BF reflects how our beliefs about the odds of a pair of hypotheses change after considering the data at hand (Lavine & Schervish, 1999; Bernardo & Smith, 2009). Note that in this paper, we always assume that both hypotheses are equally likely a priori such that $\frac{P(\mathcal {H}_0)}{P(\mathcal {H}_1)}=1$.

### The Bayes factor versus *p* values

The BF is the most prominent tool for hypothesis evaluation and model selection in the Bayesian framework (Kass & Raftery, 1995) and a viable alternative to *p* values as it does not suffer from the pitfalls of frequentist inference (Hoijtink, Klugkist, & Boelen, 2008), some of which will be mentioned here. First, the interpretation of the BF is simple and intuitive. As a quantification of evidence for a certain hypothesis in comparison to another, $BF_{01}=5$ means that the data supports $\mathcal {H}_0$ five times more than $\mathcal {H}_1$. Conversely, $BF_{01}=0.2$ means that the data supports $\mathcal {H}_1$ five times more than $\mathcal {H}_0$. Contrarily, widespread misinterpretations of *p* values persist in social and psychological science, resulting in incorrect or flawed inferences (Wagenmakers, Lee, Lodewyckx, & Iverson, 2008).

Second, BFs can provide evidence *in favor* of the null hypothesis (Hoijtink et al., 2019; Keysers, Gazzola, & Wagenmakers, 2020), while in the framework of NHST, $\mathcal {H}_0$ can only be rejected but never accepted.

Third, in the presence of minuscule effects, Bayesian hypothesis evaluation does not tend to reject the null in huge samples (Hoijtink, van Kooten, & Hulsker, 2016) as is the case in NHST (Lantz, 2013), often leading to overconfident conclusions (Kaplan et al., 2014). Instead, they simply reflect the magnitude of cumulative evidence for the best hypothesis under consideration with increasing precision as additional data are analyzed.

Fourth, as shown in Eq. 10, the probability of a hypothesis being true given the data $P(\mathcal {H} \mid X)$ can be calculated. Contrarily, in NHST one can only compute the probability of finding the data at hand or more extreme data, given that $\mathcal {H}_0$ is true, $P(X \ge x \mid \mathcal {H}_0)$. Researchers typically strive to learn about the former quantity rather than the latter because they are interested in the probability of their hypothesis being correct rather than the probability of finding their results (or more extreme ones) given $\mathcal {H}_0$ (Gill, 1999). Simulation studies have shown that in many scenarios, the correlation between $P(X \ge x \mid \mathcal {H}_0)$ and $P(\mathcal {H} \mid X)$ is quite low (Trafimow & Rice, 2009), suggesting that knowing one does not necessarily give insight into the other. The only scenario where $P(\mathcal {H} \mid X \ge x)=P(X \ge x \mid \mathcal {H})$ is when $P(\mathcal {H})=P(X \ge x)$, and there is usually no justification to assume this equality (Gill, 1999). Worse still, many researchers are convinced to have obtained $P(\mathcal {H} \mid X)$ after inspecting *p* values, resulting in incorrect interpretations of results and inferential errors (Meehl, 1990; Hubbard, 2011).

Finally, as BFs do not rely on controlling type I error rates, they can be recomputed as more data are being analyzed without needing to be corrected for multiple testing, a procedure which is referred to as “Bayesian sequential design” or “Bayesian updating” (Moerbeek, 2021, 2023). One could even argue that this method can be used *instead of* of SSD because one can just stop collecting more data once a certain BF is achieved (Schönbrodt, Wagenmakers, Zehetleitner, & Perugini, 2017; Heck et al., 2022). However, in the case of longitudinal designs as well as small population sizes (such as in rare diseases), updating is not suitable (Fu et al., 2021). This is because, especially when the duration of the intervention is long, adding a new wave of participants to the sample would require a significant amount of additional resources and might substantially increase the study’s duration. Additionally, ethical committees and funding agencies often require an a priori justification of sample size, which can only be done via SSD.

**Table 1:** Arguments to the function BayeSSD

Argument	Type	Meaning	Default value
eta	numeric	$\eta$, the desired power level for both hypotheses	0.8
BFthres	numeric	$BF_{thres},$ the threshold a BF must exceed in order to be considered convincing	3
eff.size	numeric	$\delta ,$ the expected standardized effect size	0.8
m	numeric	number of datasets to be simulated in each iteration under each hypothesis	1000
t.points	vector	$T_j,$ the time points of the measurement occasions, equal and unequal spacing possible	c(0,1,2,3,4)
beta1	numeric	specify the main effect of *time* in the control condition	0
log	logical	use log-linear growth?	FALSE
var.u0	numeric	$\sigma ^2_{u0},$ the intercept variance	0.03
var.u1	numeric	$\sigma ^2_{u1},$ the slope variance	0.1
var.e	numeric	$\sigma ^2_e,$ the residual variance	0.02
cov	numeric	$\sigma _{u0u1},$ the covariance between intercept and slope variance	0
fraction	numeric	*J*, fraction of information to specify prior: $b = J/N_{eff}$	1
sensitivity	logical	execute sensitivity analysis for different values (1, 2, 3) for fraction?	FALSE
hyp	string	perform SSD for which hypothesis, “h0”/“H0”, “h1”/“H1” or “both”/“b”	"both"’/"b"’
test	string	which hypothesis should be compared against? If “alt”, then $\mathcal {H}_0$ vs. $\mathcal {H}_1$ or vice versa, if “Hc”/“hc”, then against the complement hypothesis ($\mathcal {H}_c$), if “Hu”/“hu”, then against the unconstrained hypothesis ($\mathcal {H}_u$)	"alt"’
seed	numeric	set a seed for reproducibility	NULL

Default values are based on Raudenbush et al. (2001)

### Hypotheses

The traditional NHST approach to formulating hypotheses is to postulate a null hypothesis ($\mathcal {H}_0$) stating that “nothing is going on” and an alternative hypothesis ($H_1$) which usually reflects the researcher’s expectations. Upon confirming that the theoretical probability of the obtained results under $\mathcal {H}_0$ is low enough ($p<.05$) and therefore rejecting $\mathcal {H}_0$, the researcher concludes that $H_1$ is true (Perezgonzalez, 2015). As we have already commented on the drawbacks of this inferential procedure earlier in this section, we will now focus on the different types of hypotheses we consider in this paper.

In most clinical intervention studies, the researcher has some expectation as to which treatment intervention works better than the other. As pointed out in the section on the multilevel model, the parameter of interest is $\beta _2$, representing the interaction effect between time and treatment condition. If the researcher expects the treatment condition (coded as $C_i=1$) to perform better than the control (coded as $C_i=0$), then the research hypothesis is $\mathcal {H}_1:\beta _2>0$. In the context of Bayesian hypothesis evaluation, the research hypothesis can be compared against various other hypotheses, one of which is the null hypothesis $\mathcal {H}_0: \beta _2=0$. Another option is the complement hypothesis, which includes all the parameter space left uncovered by the research hypothesis $\mathcal {H}_c: not \; \mathcal {H}_1$. In this case, the complement hypothesis would be $\mathcal {H}_c: \beta _2\le 0$. Alternatively, $\mathcal {H}_1$ can be compared against the unconstrained hypothesis $\mathcal {H}_u: \beta _2$. This hypothesis is the most general and the least specific, as it allows any value in the parameter space for $\beta _2$. The unconstrained hypothesis can be looked upon as a “fail safe hypothesis” and is particularly useful when comparing a whole set of hypotheses with each other (Hoijtink et al., 2019). Comparing $\mathcal {H}_1$ against $\mathcal {H}_c$ or $\mathcal {H}_u$ is practical because the entire parameter space is covered and unexpected results are accounted for. On the other hand, many applied researchers are more used to comparing their research hypothesis against a null hypothesis, so we made this the default in our function, with the possibility to compare against $\mathcal {H}_c$ or $\mathcal {H}_u$ via the “test” argument (see Table 1).

### The approximate adjusted fractional Bayes factor

A number of different approaches to calculating the BF can be found in the literature but when evaluating informative hypotheses about multilevel model parameters, the *approximate adjusted fractional Bayes factor* (henceforth abbreviated as *BF*) stands out for its convenience and straightforward computation (Mulder, 2014; Gu et al., 2018). While Gu, Mulder, and Hoijtink (2018) provide a more extensive overview of this way of calculating the BF, we limit ourselves to briefly describing its main characteristics. Note that despite providing valuable insight into the calculation of the BF, the information contained in this subsection is not a strict necessity for using our function. For the less technical reader, it may suffice to be able to interpret the BF along with the results of the Bayesian SSD.

As shown by Mulder, Hoijtink, and Klugkist (2010), Eq. 9 for the BF of a hypothesis *i* against the unconstrained hypothesis $\mathcal {H}_u$ can be rewritten as

11 $$\begin{aligned} BF_{iu}=\frac{fit_i}{comp_i} \end{aligned}$$

and the BF between two competing hypotheses $\mathcal {H}_i$ and $\mathcal {H}_i'$ can be expressed by

12 $$\begin{aligned} BF_{ii'}=\frac{fit_i/comp_i}{fit_{i'}/comp_{i'}}, \end{aligned}$$

where $fit_i$ is the relative fit and $comp_i$ the relative complexity of hypothesis *i* (Gu et al., 2018). Note that for a hypothesis with only equality constraints, expression (11) is equal to the so-called Savage–Dickey ratio (Dickey, 1971; Wagenmakers, Lodewyckx, Kuriyal, & Grasman, 2010).

Fit and complexity are calculated using the prior and posterior distributions concerning the parameter of interest. The prior distribution generally reflects the a priori belief about the parameter, and the posterior distribution summarizes the information from the prior and the data. The complexity of hypothesis *i* is defined as the proportion of the parameter space under $\mathcal {H}_i$ that is in accordance with the prior, indicating how general the predictions of $\mathcal {H}_i$ are. The more general (i.e., less specific) the prediction, the higher the complexity. Fit, on the other hand, indicates how well $\mathcal {H}_i$ describes the data, being defined as the proportion of the parameter space under $\mathcal {H}_i$ that is in accordance with the posterior. If the fit of both hypotheses is equal, the BF will prefer the least complex hypothesis, acting as an Occam’s razor (Hoijtink et al., 2019).

In this particular way of computing the BF, a fraction of the data ($X^b$) is used to construct a default prior. This means that the researcher does not need to specify the distributional form of the prior (O’Hagan, 1995) and the subjective input from the researcher is only expressed in the form of the specified hypotheses and the choice of the size of $X^b$. This addresses a common critique stating that BFs are too sensitive to the prior specified by the researcher, and applied researchers are often uncertain about how to construct said prior (Frick, 1996; Trafimow, 2003).

**Fig. 1:**
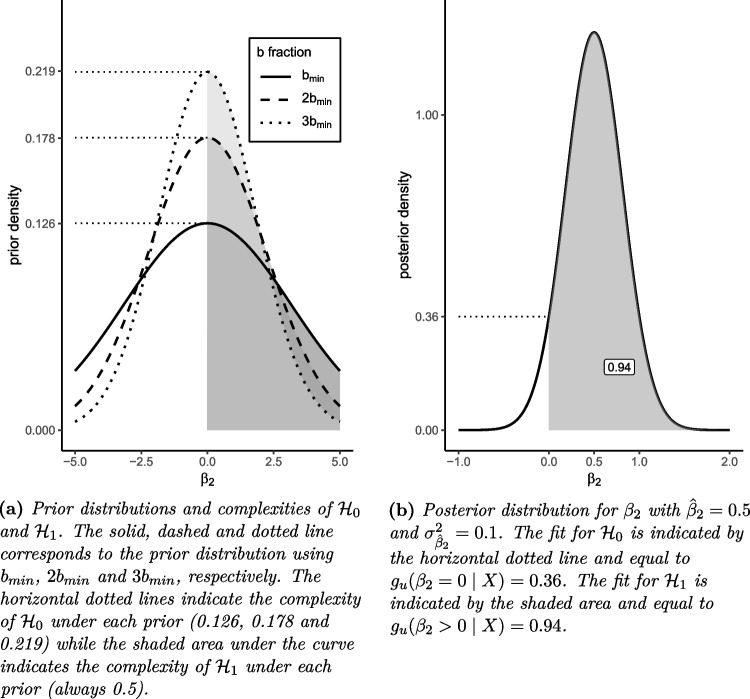
Prior and posterior distributions with fit and complexity for $\mathcal {H}_0$ and $\mathcal {H}_1$, respectively

Furthermore, a normal distribution is used for the prior and as an approximation of the posterior. The prior distribution is adjusted such that it is centered around the boundary of the parameter space constrained by the hypotheses at hand. In both our examples of the psychological treatment intervention studies with hypotheses $\mathcal {H}_0$ (6) and $\mathcal {H}_1$ (7), the focal point and thus the center of the prior is zero. The unconstrained prior $h_u(\beta _2 \mid X^b)$ and posterior $g_u(\beta _2 \mid X)$ are then approximated by

13 $$\begin{aligned} h_u(\beta _2 \mid X^b) \approx N(0, \sigma ^2_{\hat{\beta }_2} / b) \end{aligned}$$

and

14 $$\begin{aligned} g_u(\beta _2 \mid X) \approx N(\hat{\beta }_2, \sigma ^2_{\hat{\beta }_2}), \end{aligned}$$

respectively. The data at hand are denoted by *X* and the fraction of the data used to inform the prior is denoted by $X^b$. The maximum likelihood estimate of our parameter of interest is $\hat{\beta }_2$ and its squared standard error is $\sigma ^2_{\hat{\beta }_2}$. The formulae for the fit of the equality constrained hypothesis $\mathcal {H}_0$ and of the inequality constrained hypothesis $\mathcal {H}_1$ are therefore

15 $$\begin{aligned} fit_0=g_u(\beta _2 = 0 \mid X) \end{aligned}$$

and

16 $$\begin{aligned} fit_1=\int _{\beta _2>0}g_u(\beta _2 \mid X)d\beta _2, \end{aligned}$$

respectively. Finally, the complexities of hypotheses $\mathcal {H}_0$ and $\mathcal {H}_1$ are given by

17 $$\begin{aligned} comp_0=h_u(\beta _2=0 \mid X^b) \end{aligned}$$

and

18 $$\begin{aligned} comp_1=\int _{\beta _2>0}h_u(\beta _2 \mid X^b)d\beta _2, \end{aligned}$$

respectively. Thus, the BFs for $\mathcal {H}_0$ and $\mathcal {H}_1$ as formulated in Eqs. 6 and 7 against the unconstrained hypothesis $\mathcal {H}_u$ can be expressed analytically as

19 $$\begin{aligned} BF_{0u}=\frac{g_u(\beta _2 = 0 \mid X)}{h_u(\beta _2=0 \mid X^b)} \end{aligned}$$

and

20 $$\begin{aligned} BF_{1u}=\frac{\int _{\beta _2>0}g_u(\beta _2 \mid X)d\beta _2}{\int _{\beta _2>0}h_u(\beta _2 \mid X^b)d\beta _2}. \end{aligned}$$

As mentioned before, the fraction *b* determines the amount of data used to specify the default prior. The posterior and thus the fit of a hypothesis is unaffected by *b*. A typical choice for this quantity is the minimal

21 $$\begin{aligned} b_{min}=\frac{J}{N_{eff}}, \end{aligned}$$

where *J* is the number of independent constraints in the hypotheses under investigation and $N_{eff}$ is the effective sample size (Hoijtink, Gu, & Mulder, 2019). In our case $J=1$ because we constrain only one model parameter. In the case of $b_{min}$, the minimal number of observations necessary to identify the parameter(s) of interest is used for the prior. This method is inspired by the principle of the minimal training sample (O’Hagan, 1995; Berger & Pericchi, 1996) and has the advantage that the amount of data used to construct the prior is minimized while the part of the data used to calculate the BF is maximized.^2^ Alternative choices for *b* in our function are $2b_{min}$ and $3b_{min}$ (see Fu et al., 2021), which generally result in a smaller prior variance and reduce the sensitivity of the BF to the prior distribution (O’Hagan, 1995; Conigliani & O’Hagan, 2000). The choice of *b* can have non-trivial consequences for the resulting BF for hypotheses with (about-) equality constraints, as will be shown in the following example. Suppose that after the collection of data of $N=100$ individuals and analysis using multilevel regression, the estimate for the parameter is $\hat{\beta }_2=0.5$ with a corresponding squared standard error of $\sigma ^2_{\hat{\beta }_2}=0.1$. We now want to test the hypotheses (6) and (7) against each other using the BF. Figure 1 depicts the resulting complexities and fits for hypotheses (6) and (7), respectively. The influence of *b* on the complexity of equality constrained hypotheses becomes apparent in Fig. 1: The complexity of $\mathcal {H}_0$ with $b_{min}$ is 0.126. When using $2b_{min}$, the complexity increases to 0.178 and finally to 0.219 when using $3b_{min}$. Note that the complexity for the inequality constrained $\mathcal {H}_1$ stays the same because in both cases, the integral from zero to infinity is equal to 0.5 regardless of the prior variance. This change in complexity of $\mathcal {H}_0$ can result in substantial changes of the resulting BF: The BF of $\mathcal {H}_0$ versus the unconstrained hypothesis ($\mathcal {H}_u$) is $BF_{0u}=fit_0/comp_0=.36/.126=2.86$ when using $b_{min}$ but only $BF_{0u}=0.36/0.178=2.02$ when using $2b_{min}$ and $BF_{0u}=0.36/0.219=1.64$ when using $3b_{min}$. This phenomenon also presents itself when inspecting the BF for $\mathcal {H}_0$ versus $\mathcal {H}_1$. The BF of $\mathcal {H}_0$ versus $\mathcal {H}_1$ is $BF_{01}=BF_{0u}/BF_{1u}=2.86/1.88=1.52$ when using $b_{min}$ but only $BF_{0u}=1.07$ when using $2b_{min}$ and only $BF_{0u}=0.87$ when using $3b_{min}$. This means that in some cases, the BF prefers a different hypothesis over the other depending on which *b* is chosen. More specifically, equality-constrained hypotheses such as $\mathcal {H}_0$ are more preferred by the BF when using smaller *b* and less preferred with larger *b* (Gu et al., 2018). Consequently, one needs to be aware of this sensitivity when choosing *b*. Gu et al. 2018 and Hoijtink 2019 provide some guidelines on which *b* to choose in a specific situation. In our example, $b_{min}$ would be an appropriate choice because of the small sample size ($N=100$). This is because we want to minimize the amount of data used to construct the prior. On the other hand, when the sample size is large and robustness against a potentially misspecified prior is of importance, a larger *b* may be a more suitable option (O’Hagan, 1995). There are other ways to specify *b*, for example $b_{freq}$, which results in equal type I and type II error probabilities (Gu, Hoijtink, & Mulder, 2016; Hoijtink, 2022), but these are beyond the scope of this article.

As illustrated in the above example, the BF is completely independent of *b* when the hypotheses at hand contain no (about-) equality constraints (Mulder, 2014). This is because the complexity (defined as the integral of the prior between the bounds of the parameter space under the hypothesis) stays the same regardless of the shape of the prior (Hoijtink et al., 2019). In this paper, we consider the consequences of some choices for *b* through an optional sensitivity analysis. Similar to the function by Fu et al. (2021), the result of the SSD is reported for three different choices of *b* ($b_{min}$, $2b_{min}$, and $3b_{min}$) when setting the argument sensitivity = TRUE.

## Bayesian sample size determination

To perform SSD in the Bayesian framework, one first needs to specify the degree of evidence that is considered compelling. This is done by defining $BF_{thres}$, the threshold value that the BF must exceed to be considered substantial. Although some authors suggest general cut-off values to interpret the strength of relative evidence indicated by the BF (Kass & Raftery, 1995), we recommend avoiding the fallacy of a global dichotomous decision rule, and rather letting the relative evidence for each hypothesis speak for itself (Hoijtink et al., 2019). However, in the case of SSD, one needs to specify some sort of decision rule in order to calculate the probability of making the correct inferential decision. We therefore let the researcher decide the magnitude of relative evidence that they find compelling by specifying $BF_{thres}$ prior to executing the SSD. This threshold value may be lower (e.g., 3) for more exploratory research and higher (e.g., 10) for high-stakes research.

Next, the desired power level $\eta$ needs to be established. This reflects the probability of obtaining a BF larger than $BF_{thres}$ in favor of the true hypothesis (Fu et al., 2021). This is similar to the frequentist definition, which was given previously. However, the difference is that if $\mathcal {H}_0$ is true, we also require the BF in favor of $\mathcal {H}_0$ to be at least $BF_{thres}$ with a probability of at least $\eta$. This is a crucial contrast to frequentist power as we are dealing with two conditional probabilities instead of one. These are a) $\eta _0=P(BF_{01}>BF_{thres} \mid \mathcal {H}_0)$, the chance of finding a BF favoring $\mathcal {H}_0$ which exceeds the threshold value, given that $\mathcal {H}_0$ is true and b) $\eta _1=P(BF_{10}>BF_{thres} \mid \mathcal {H}_1)$, the chance of finding a BF favoring $\mathcal {H}_1$ exceeding the threshold value, given that $\mathcal {H}_1$ is true. This discrimination between $\eta _0$ and $\eta _1$ arises because in Bayesian hypothesis testing, both $\mathcal {H}_0$ and $\mathcal {H}_1$ can be accepted and are regarded as “equals” (Hoijtink et al., 2019; Tendeiro & Kiers, 2019). Considering that most ethics and funding committees require one single value to indicate statistical power, we by default combine the two probabilities into $\eta =min(\eta _0, \eta _1)$, where $\eta$ indicates the lower bound of power for all hypotheses under consideration, similar to Wang and Gelfand (2002). However, it is possible to perform SSD for only one of the two hypotheses via the hyp argument (see Table 1), reducing the necessary computing time to a minimum.

**Fig. 2:**
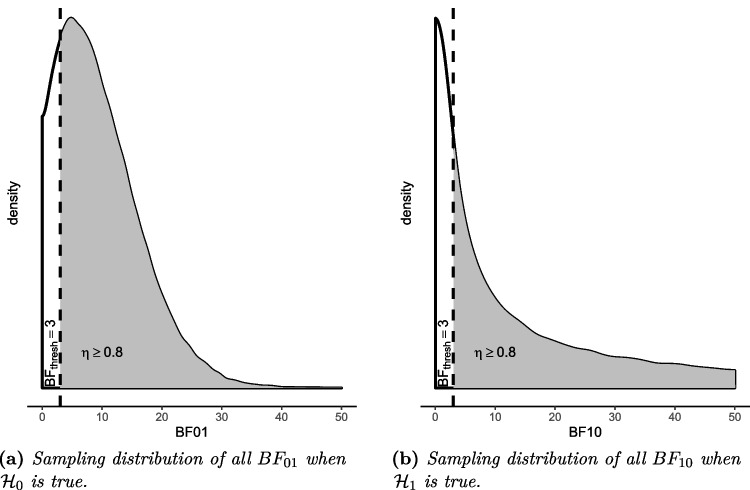
Sampling distributions $BF_{01}$ and $BF_{10}$ under $\mathcal {H}_0$ and $\mathcal {H}_1$ when the power criterion of $\eta =.80$ is met with $BF_{thres}=3$

Because it is not known how to derive the sample size resulting in a certain $\eta$ analytically for the AAFBF, we resort to simulation. To do that, we need to operationalize $\eta$ in a way that we can assess in the simulation. We therefore define $\eta$ as the proportion of BFs favoring the true hypothesis greater than $BF_{thres}$ across all simulated data sets. If the desired power level is, say, .80, then we look for the sample size for which at least 80% of the BFs favoring the true hypothesis exceed $BF_{thres}$. Figure 2 illustrates the sampling distributions of the BFs when the power criterion of $\eta =.80$ is met. It can be seen that for both hypotheses, the proportion of BFs exceeding $BF_{thres}$ is at least .80. The steps of the algorithm to execute the SSD are elaborated in the following section.

**Fig. 3:**
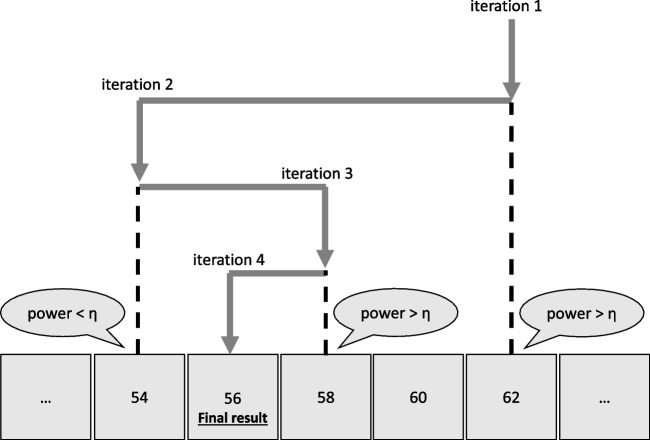
Procedure of a binary search SSD algorithm with a final result of N = 56, where $\eta$ is the desired power level

### The algorithm

The function BayeSSD was created using R version 4.2.2 (R Core Team, 2013) and the packages “lme4” version 1.1-31 (Bates, Mächler, Bolker, & Walker, 2015), “MASS” version 7.3-58.2 (Venables & Ripley, 2002), “future” version 1.34.0 (Bengtsson, 2021), and “future.apply” version 1.11.3 (Bengtsson, 2021). The logic of the function for BayeSSD is that of a binary search algorithm. In each iteration, *m* data sets with sample size *N* are generated under each hypothesis. Next, the function evaluates whether the power condition is met, that is, the proportion of *BFs* larger than $BF_{thres}$ favoring the true hypothesis is at least $\eta$ under both hypotheses (see Fig. 2). Put differently, the power condition is met if the following inequality holds under both $\mathcal {H}_0$ and $\mathcal {H}_1$

22 $$\begin{aligned} \frac{\sum _{k=1}^{m} \mathbb {I}_{(BF_k> BF_{thres})}}{m} = P(BF_{k}>BF_{thres})&\ge \eta , \end{aligned}$$

where $\mathbb {I}_{(BF_k > BF_{thres})}$ is an indicator function that is equal to 1 if the condition in brackets is met and 0 if not. If the power for both hypotheses is equal to or larger than $\eta$, then the currently evaluated *N* meets the power criterion. Note that $BF_k$ denotes the Bayes factor in the *k*th generated dataset for either $\mathcal {H}_0$ or $\mathcal {H}_1$ against the other one or alternatively, $\mathcal {H}_0$ or $\mathcal {H}_1$ against the complement hypothesis $\mathcal {H}_c$ or against the unconstrained hypothesis $\mathcal {H}_u$.

The binary search algorithm works as follows (see Fig. 3). First, a minimum ($N_{min}=30$) and maximum sample size ($N_{max}=1000$) are specified. Next, the power for the sample size in the middle of this range ($N_{mid}=0.5*(N_{min}+N_{max})$) is evaluated. If the resulting power is at least $\eta$ under both hypotheses, then $N_{mid}$ becomes $N_{max}$ in the next iteration. If either one of the power levels is less than $\eta$, $N_{mid}$ becomes $N_{min}$ in the next iteration. When $N_{mid} = N_{min} + 1$, that is, when the difference between $N_{min}$ and $N_{max}$ is equal to one, $N_{mid}$ along with $\eta _0$ and $\eta _1$ are returned as a result of the SSD algorithm. The arguments of the function along with their default values are described in Table 1.

**Table 2:** Effects of study duration (*D*) and frequency of observation (*f*) on Bayesian power

$\varvec{\mathcal {H}}_{\varvec{1}}\varvec{:\beta _2>0}$	f					
*D*	1	2	3	4	5	6
1	−	.014	.013	.014	.013	.013
2	.054	.054	.062	.074	.085	.096
3	.136	.168	.210	.253	.288	.320
4	.267	.351	.416	.473	.519	.551
5	.422	.519	.588	.639	.670	.693
6	.556	.651	.704	.736	.758	.773
7	.657	.732	.772	.795	.806	.816
8	.726	.786	.812	.829	.834	.843

Note. Number of individuals held constant at $N=100$; number of measurement occasions $n=fD+1$. $\mathcal {H}_1$ is compared against $\mathcal {H}_0: \beta _2=0$

### Effect of number of subjects, frequency of observation, and study duration on power

In the following, we study the effect of the number of individuals (*N*), the frequency of observation (*f*), and the study duration (*D*) on power in the case of linear growth and equidistant time points using the estimates from Raudenbush and Liu (2001). The subsequent simulations are executed with m = 50000 datasets under each hypothesis in each iteration, an intercept variance of var.u0 = 0.0333, a slope variance of var.u0 = 0.0333, no covariance between the random effects (cov = 0), an error variance of var.e = 0.02, a fraction of information of fraction = 1 resulting in $b=1/N_{eff}$, a BF threshold of BFthres = 3, no treatment effect in the control group (beta1=0), and a standardized effect size of eff.size = 0.8. Thus, in the case where $\mathcal {H}_1: \beta _2>0$ is the true data-generating mechanism, $\beta _2=\delta \sqrt{\sigma _{u1}}=0.8*\sqrt{0.001}=0.0253$ (Raudenbush & Liu, 2001). On the other hand, if $\mathcal {H}_0: \beta _2=0$ is the true data-generating mechanism, $\delta =0$, obviously. Table 2 shows the effects of increasing *D* and *f* on the power of $\mathcal {H}_1$, while holding the number of subjects constant at $N=100$. Note that the results for $\mathcal {H}_0$ can be found in Appendix A as the power for $\mathcal {H}_0$ remains largely unaffected by *D* and *f*. Table 3 illustrates the effects of increasing *D* and *N* for both hypotheses while keeping the frequency of observation constant at $f=1$. Note that instead of an extensive simulation study resulting in large tables, we opt for a more contained simulation which showcases the most important operating characteristics of the power statistic in Eq. 22 in a clear and straightforward manner. This is also because we want to discourage researchers from consulting tables in place of calculating the power/necessary sample size for their specific study design. In Tables 2 and 3, we show that the Bayesian power behaves according to our expectations based on previous research on frequentist power in multilevel models (e.g., Moerbeek, 2008). For the same tables with m = 10,000 datasets in case $\mathcal {H}_1$ is compared against $\mathcal {H}_c$ or $\mathcal {H}_u$, see Appendix B.

**Table 3:** Effects of study duration (*D*) and number of individuals (*N*) on Bayesian power for $\mathcal {H}_0$ and $\mathcal {H}_1$

	*N*									
*D*	20	40	60	80	100	120	140	160	180	200
$$ \varvec{\mathcal {H}}_{\varvec{0}}$$										
2	.880	.927	.940	.950	.957	.961	.966	.968	.971	.973
3	.895	.931	.947	.957	.960	.965	.969	.970	.973	.974
4	.898	.937	.951	.958	.963	.968	.971	.972	.974	.976
5	.900	.935	.949	.958	.964	.969	.970	.974	.976	.977
6	.897	.934	.950	.958	.964	.968	.971	.973	.974	.978
7	.894	.934	.949	.958	.962	.968	.968	.971	.975	.976
8	.889	.932	.948	.955	.961	.965	.969	.971	.973	.975
$$\varvec{\mathcal {H}}_{\varvec{1}}$$										
2	.034	.036	.038	.047	.053	.059	.068	.075	.086	.096
3	.053	.066	.086	.109	.136	.163	.189	.218	.250	.280
4	.083	.119	.162	.218	.267	.323	.379	.437	.496	.542
5	.120	.184	.258	.340	.420	.500	.574	.642	.703	.755
6	.162	.246	.350	.456	.559	.643	.721	.791	.841	.877
7	.198	.309	.427	.553	.655	.744	.818	.875	.910	.940
8	.225	.350	.495	.622	.725	.811	.873	.918	.946	.967

Note. Frequency of observations held constant at $f=1$; $\mathcal {H}_0: \beta _2=0$, $\mathcal {H}_1: \beta _2>0$, number of measurement occasions $n=fD+1$

As can be seen in Tables 2 and 3, increasing one of the quantities *f*, *D*, and *N* consistently leads to higher power for $\mathcal {H}_1$. It can further be noted that increasing *N* is the most efficient strategy to improve power because the power level increases until $\eta =1$ while the effect of increasing *f* or *D* levels off at some point. This pattern is in line with previous findings on frequentist power in multilevel models (Moerbeek, 2008). Furthermore, it can be seen that for very short durations, increasing the frequency has a negligible effect on power. Only after exceeding a certain study duration (here: 2), the increased frequency of observation benefits power.

The fact that some cells in Table 2 are roughly equal to each other illustrates the possible trade-off of design factors against each other. For example, a study with $D=4$ and $f=1$ has a power of .268. If one were to aim to increase that power level to .41, then two modifications in terms of study design would be available to the researcher. The first one is to increase *f* from one to four. The second option is to keep *f* constant while increasing *D* from four to five. As can be seen in Table 2, both options result in a power level of roughly .41. However, one of the two options may be preferred because of budgetary or practical constraints. This is an example of power equivalence, a relation between two different designs that holds true if the two designs have the same power to detect a given effect (von Oertzen, 2010).

Through these simulations, we get an impression of how design factors, such as the number of measurement occasions or the study duration, can be traded off against each other while keeping a constant power level. For example, von Oertzen and Brandmaier (2013) showed that the duration of a study with a certain design can be reduced from 13 to 11.8 years without losing any power by means of increasing the number of measurement occasions from six to seven. Later, it was shown that such power equivalence also holds for Bayesian hypothesis tests (Stefan & von Oertzen, 2020) for the slope variance at the second level. Researchers planning their trials should consider these factors to achieve the most efficient (and thus, ethical) study design.

## Empirical examples

In this section, two examples are introduced to illustrate the SSD procedure. While both of these examples consist of “real” empirical longitudinal studies, we do not use their original datasets. Rather, we execute the SSD for a potential replication study with a certain desired power level. For that purpose, we use the author’s findings about estimates of fixed and random effects as “ingredients” for our SSD algorithm. The two examples represent two typical cases: a) linear growth and equidistant time points and b) log-linear growth and non-equidistant time points.

### Example 1: Antisocial thinking during adolescence: Linear growth with equally spaced measurement occasions including sensitivity analysis

Similar to Raudenbush and Liu (2001) we use the study design of the National Youth Survey (NYS; Elliott, Huizinga, & Menard, 2012) to illustrate our method. Suppose a researcher aims to replicate the findings of the NYS using the BF and wants to determine the sample size corresponding to a user-selected power level. To determine that sample size, we use the findings of the original study about parameters such as the variances of the random effects as a starting point for our simulation. In the first cohort of the NYS, adolescents in the US were interviewed yearly between 1976 and 1980 about their tolerance of antisocial behavior. Because previous findings suggest a linear effect of time on the outcome in this age (Raudenbush & Chan, 1993), we use linear growth for the SSD. Furthermore, the distance between measurement occasions is constant, resulting in an equally spaced time vector. For a detailed description of the data, the interested reader is referred to Miyazaki and Raudenbush (2000).

Because in the original study there were no treatment and control conditions, we add those to the design in order to make our method applicable to this example, analogous to Raudenbush and Liu (2001). We suppose that in the treatment condition, adolescents are educated about the consequences of antisocial behavior, while this is not the case in the control condition. Our aim is to determine whether the rate of decline in the outcome (tolerance of antisocial behavior) differs significantly between the treatment and control conditions. The corresponding hypotheses are $\mathcal {H}_0: \beta _2=0$ and $\mathcal {H}_1:\beta _2>0$, as in the previous section.

The $n=5$ measurement occasions are equally spaced, resulting in a time vector of $T_j=(0,1,2,3,4)^\intercal$ in years. Decline over time is assumed to be linear, so the argument log is set to FALSE when running the function. Using the estimates from Raudenbush and Liu (2001), we can fill in the expected variance of the residuals, intercept, and slope: $\sigma ^2_e=0.0262$, $\sigma ^2_{u0}=0.0333$, $\sigma ^2_{u1}=0.0030$. The expected standardized effect size is $\delta =0.40$ corresponding to an expected coefficient of interaction $\beta _2=\delta \sqrt{\sigma ^2_{u1}}=0.0219$. We assume the treatment effect in the control group to be zero so we set beta1=0. As we did not find the value for the covariance between the random effects, we assume that it is equal to zero. We decide to test our hypothesis $\mathcal {H}_1$ against the null hypothesis $\mathcal {H}_0$, so we set the test argument to "alt". Next, we select a threshold value $BF_{thres}=3$ and the desired power $\eta =.80$. Also, we ask for a sensitivity analysis for different values for the *b* fraction. Note that because the SSD procedure is a stochastic process, we need to set a seed for reproducibility. Now, we have all the ingredients for the SSD and feed them to the function as follows:
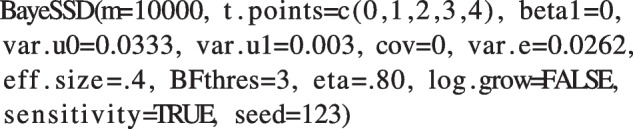


The following output informs the user about the results of the SSD procedure. Note that the resulting power is not always exactly equal to $\eta$ but rather the power level closest to it out of all considered sample sizes.
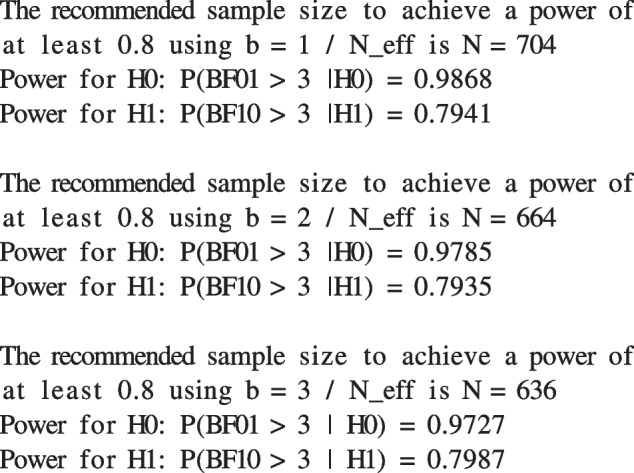


The output of the function informs us that we need to sample at least $N_1=704$ participants to achieve an at least 80% chance of making the correct inferential decision when using $b_{min}=\frac{1}{N_{eff}}$, $N_{2}=664$ participants when using $2b_{min}=\frac{2}{N_{eff}}$ and $N_{3}=636$ participants when using $3b_{min}=\frac{3}{N_{eff}}$. Furthermore, we learn that the choice of the *b* fraction significantly influences the resulting power. When using $b_{min}$, we need 40 more participants to achieve the desired power level compared to $2b_{min}$ and 68 more compared to $3b_{min}$. As mentioned before, the choice of *b* depends on whether a minimally informed prior ($b_{min}$) or a robust prior ($2b_{min}$, $3b_{min}$) is preferred. The choice should *not* be determined by the desire to use a smaller sample size. For a more extensive overview and guidelines for choosing *b*, see Gu et al. (2018).

The above sample sizes may be considered high by the researcher and it is possible that available resources are insufficient for that large of a sample. As can be seen in Tables 2 and 3, there are options to obtain more power other than increasing *N*. One might consider increasing the study duration *D* or the frequency of observation *f* in order to achieve similar power with a smaller *N*.

### Example 2: Systematic patient feedback in psychotherapy: Log-linear growth and unequally spaced measurement occasions

For our second example, we use the study by Bovendeerd, De Jong, De Groot, Moerbeek, M. and De Keijser (2022) as a starting point. In this study, patients were allocated to either a treatment condition (therapy with feedback) or a control condition (therapy without feedback) and assessed at four occasions (week 0, 5, 13, and 638) with the Outcome Questionnaire (OQ-45; Lambert, Gregersen, & Burlingame, 2004). While it was the intention of the authors to measure every participant at the same occasion, this was not the case in practice. For the sake of illustrating our method for SSD, however, we assume that all subjects will be measured at the same points in time in the replication study. The data have a three-level structure (measurement, patient, therapist). Note that the amount of variance at the therapist level was very small as compared to the patient and measurement level. In order to illustrate our method in a simple, straightforward manner, we omit the third level (therapist) so that observations are nested only within patients. Again, we suppose that we perform SSD for a replication study and use the findings of Bovendeerd et al. (2022) as a starting point. The spacing between the measurement occasions is *unequal* and time is assumed to have a *log-linear* effect on the outcome.

In line with the results found by the authors, we expect that in the treatment condition, patients improve faster compared to the control condition (therapy without feedback). Note that a *lower* score on the outcome (OQ-45) corresponds with *more* well-being and *less* psychiatric symptoms (Bovendeerd et al., 2022). Therefore, we expect the growth to be smaller in the treatment group compared to the control group. Suppose that in this case, we want to compare our hypothesis to its complement $\mathcal {H}_c$, resulting in the following hypotheses:

23 $$\begin{aligned} \mathcal {H}_1: \beta _2 < 0 \end{aligned}$$

24 $$\begin{aligned} \mathcal {H}_c: \beta _2 \ge 0 \end{aligned}$$

In this case, the $n=4$ measurement occasions are irregularly spaced in time. Individuals were measured before, during, and after treatment. Additionally, growth over time is assumed to be log-linear, meaning that the resulting time vector is $T_j'=log(0, 5, 13, 638)$. We therefore set the log argument to TRUE and the t.points argument to c(0, 5, 13, 638). Previous findings of Bovendeerd et al. (2022) provide us with the necessary estimates for our function: $\sigma ^2_e=172,191$, $\sigma ^2_{u0}=237.114$, $\sigma ^2_{u1}=13.234$, $\sigma _{u0u1}=18.282$. The authors’ estimate of the interaction coefficient is $\beta _2=-2.051$ corresponding to a standardized effect size of -0.564. Note that because $\mathcal {H}_1: \beta _2<0$ instead of $\mathcal {H}_1: \beta _2>0$, we have to reverse the sign (direction) of the effect size such that $\delta =0.564$. This is simply because our function does not accept negative effect sizes. However, this reversal does not influence the result of the SSD because testing $\beta _2<0$ with $\delta =-0.564$ is equivalent to testing $\beta _2>0$ with $\delta =0.564$. We further assume the treatment effect in the control group to be zero so we set beta1=0. Because we decide to test our hypothesis $\mathcal {H}_1$ against its complement hypothesis $\mathcal {H}_c$, we set the test argument to "Hc". Also, because we are not interested in the power for $\mathcal {H}_0$, we can set the argument hyp to "H1", reducing the computation time. Next, we establish a threshold value $BF_{thres}=10$ and the desired power $\eta =.70$. Now, we have all the ingredients for the SSD and feed them to the function as follows:
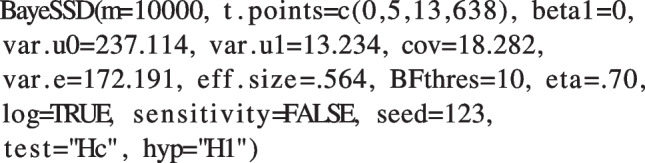


The following output informs the user about the results of the SSD procedure.
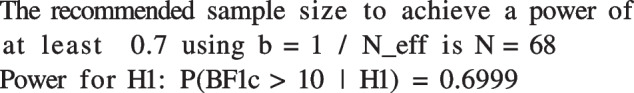


This means that if $\mathcal {H}_1$ is indeed true, we need to sample at least $N=68$ participants to achieve a probability of at least 70% obtain a $BF_{1c}$ of at least 10 when using $b=\frac{1}{N_{eff}}$.

## Conclusion

In this paper, we present a method for performing SSD in Bayesian multilevel analysis with linear or log-linear growth using Monte Carlo simulation. This method is implemented in the open-source R function BayeSSD. With this function, researchers can perform Bayesian SSD tailored to their specific longitudinal trial and use the results to motivate their sample size in proposals for funding agencies and ethics committees. To limit the intense computational cost of running the simulation within the function, we a) developed a binary search algorithm that requires relatively few iterations, similar to Fu et al. (2021) and b) parallelized the code such that the simulation runs on multiple cores. We demonstrate the use of this function using two empirical examples from psychological longitudinal intervention studies. Furthermore, we demonstrate that the algorithm’s operating characteristics are clear-cut and align with general expectations. The simulation results presented in this paper highlight that the basic concepts of frequentist power in multilevel models also apply in Bayesian power in these models: Increasing the number of level 2 observations (*N*) boosts power up to $\eta =1$ whereas increasing the number of level 1 observations (*n*) raises the power only up to a certain point. Increasing the duration (*D*) of a study also boosts power, a finding that aligns with the results by Raudenbush and Liu (2001). However, in practice, it is important to examine the associated costs as well as consequences for attrition, which is a common occurrence in psychological longitudinal intervention studies. Longer studies with more measurement occasions tend to result in higher drop-out rates, resulting in a loss of power (Moerbeek, 2008). Trade-offs like these should be taken into account in the design phase of a study, and further studies on Bayesian SSD taking attrition into account are needed. In order to provide the users of our function with some guidance as to which input values should be chosen, we developed a ShinyApp that visualizes the impact of choosing certain combinations of parameter values on the resulting regression slopes. Additionally, researchers can consult experts and/or findings from previous studies. For example, Moerbeek and Teerenstra 2015 provide an extensive overview of studies that summarize many estimates of the ICC in cluster randomized trials as found in the literature. Similarly, various estimates of the ICC can be found in the Clustered Outcomes Dataset (CLOUD) bank (Korevaar et al., 2021). Something of the like is also needed for longitudinal data to enable researchers to make more informed estimates about their expected (co-)variance components and model coefficients.

The novelty of this research lies in the methods employed: Multilevel models are a flexible and viable method to evaluate the effectiveness of treatments longitudinal intervention studies. They have notable advantages over alternative approaches, such as repeated measures ANOVA which have been highlighted in the introduction. The BF with its relatively straightforward interpretation is a versatile tool for evaluating hypotheses without relying on the often misinterpreted *p* values. Additionally, it provides one with the possibility of obtaining evidence *in favor* of the null hypothesis, something that is not possible within NHST. The specific way of calculating the Bayes factor via the AAFBF (Gu et al., 2018) does not require the researcher to specify a prior distribution, thereby removing some subjectivity from the inferential procedure and making the method more accessible for researchers who are less experienced in Bayesian statistics. Furthermore, calculating the AAFBF is much less computationally intensive compared to other BFs, which employ complicated MCMC sampling algorithms to sample from the posterior. The Bayesian approach to statistical power generally enables us to move away from the unilateral approach of NHST methods and provides a new perspective on the concept of power.

However, the method presented in this study has limitations. The R function is currently unable to handle more than two informative hypotheses or more than one regression parameter at a time. In the subsequent project, these limitations will be addressed along with the inclusion of a survival function to take different patterns of attrition into account when calculating power. Another interesting expansion of our method would be within-person interventions where individuals switch treatment conditions over time, as well as the optimal allocation of measurement occasions in time.

To our knowledge, this is the first study introducing an open-source software for Bayesian SSD in longitudinal trials using frequentist estimates. We hope that these results along with the R function BayeSSD accessible on GitHub enable researchers who use Bayesian hypothesis evaluation with multilevel models to carry out SSD in a straightforward manner.

## Data Availability

Data sharing is not applicable to this article as no empirical data was collected or used.

## Appendix A: Table with effects of study duration and frequency of observation on Bayesian power for H0

**Table 4:** Effects of study duration (*D*) and frequency of observation (*f*) on Bayesian power

$\varvec{\mathcal {H}}_{\varvec{0}}\varvec{:\beta _2=0}$	*f*					
*D*	1	2	3	4	5	6
1	$$ -$$	.961	.965	.971	.972	.974
2	.957	.969	.974	.976	.978	.980
3	.961	.971	.975	.977	.980	.980
4	.964	.971	.975	.978	.978	.979
5	.963	.972	.974	.977	.977	.978
6	.964	.969	.972	.974	.974	.976
7	.962	.969	.972	.972	.972	.972
8	.964	.968	.968	.968	.970	.971

*Note.* Number of individuals held constant at $N=100$; number of measurement occasions $n=fD+1$. $\mathcal {H}_0$ is compared against $\mathcal {H}_1: \beta _2>0$

## Appendix B: Tables with effects of study duration and frequency of observation when comparing against Hc and Hu

**Table 5:** $$\varvec{\mathcal {H}}_{\varvec{0}}\varvec{:\beta _2=0} \text { vs. } \varvec{\mathcal {H}}_{\varvec{c}} \varvec{=} \varvec{\mathcal {H}}_{\varvec{u}}\varvec{:} \varvec{\beta }_{\varvec{2}}$$

*f*						
*D*	1	2	3	4	5	6
1	−	.962	.969	.974	.975	.975
2	.960	.971	.977	.980	.980	.982
3	.961	.970	.976	.978	.982	.977
4	.966	.971	.978	.981	.980	.979
5	.967	.973	.976	.976	.977	.980
6	.966	.969	.972	.972	.976	.977
7	.966	.970	.974	.975	.975	.976
8	.966	.969	.968	.970	.975	.972

*Note.* Number of individuals held constant at $N=100$; number of measurement occasions $n=fD+1$. Note that the complement of $\mathcal {H}_0$ is equal to the unconstrained $\mathcal {H}_u$

**Table 6:** $$\varvec{\mathcal {H}}_{\varvec{1}}\varvec{:\beta _2>0} \text { vs. } \varvec{\mathcal {H}}_{\varvec{c}}\varvec{: \beta _2<0}$$

*f*						
*D*	1	2	3	4	5	6
1	−	.447	.455	.475	.484	.501
2	.647	.694	.741	.776	.801	.824
3	.827	.881	.913	.938	.953	.963
4	.923	.960	.976	.986	.990	.991
5	.970	.985	.993	.996	.996	.996
6	.986	.994	.997	.997	.998	.998
7	.992	.996	.999	.998	.999	.999
8	.996	.998	.999	.999	1.000	.999

*Note.* Number of individuals held constant at $N=100$; number of measurement occasions $n=fD+1$

**Table 7:** $$\varvec{\mathcal {H}}_{\varvec{1}}\varvec{:\beta _2>0} \text { vs. } \varvec{\mathcal {H}}_{\varvec{u}}\varvec{:} \varvec{\beta }_{\varvec{2}}$$

*f*						
*D*	1	2	3	4	5	6
1	−	.231	.232	.254	.252	.269
2	.409	.454	.511	.555	.595	.629
3	.638	.714	.773	.831	.855	.882
4	.798	.875	.912	.941	.956	.963
5	.899	.944	.968	.975	.980	.985
6	.944	.973	.982	.984	.989	.991
7	.969	.984	.989	.990	.994	.994
8	.977	.989	.991	.993	.994	.996

*Note.* Number of individuals held constant at $N=100$; number of measurement occasions $n=fD+1$. The Threshold for the BF in this case is set to 1.8 as $BF_{1u}$ has an upper bound of 2

## Footnotes

1 In some cases, it is possible that $N_{eff}>N_{total}$, see Ferrer & Vallejos 2025.

2 Note that two different definitions of the minimal *b* can be found in the literature. While Gu et al. (2018) suggest $b_{min}=\frac{J+1}{N_{eff}}$, we use the $b_{min}$ by Hoijtink et al. 2019 which is also implemented in the R-packages *bain* (Gu, Hoijtink, Mulder, & van Lissa,^2021^) and *BFpack* (Mulder et al., 2021).

## Code Availability

The entire R code containing the functions necessary to carry out the sample size determination algorithm as well as the code used for the creation of figures and tables can be found on the personal GitHub repository of the first author: https://github.com/ulrichlosener/BayesianSSD.
